# Environmental Toxicants and Preterm Birth: A Bibliometric Analysis of Research Trends and Output

**DOI:** 10.3390/ijerph19052493

**Published:** 2022-02-22

**Authors:** Manuel S. Vidal, Ramkumar Menon, Gracia Fe B. Yu, Melissa D. Amosco

**Affiliations:** 1 College of Medicine, University of the Philippines Manila, Manila 1000, Philippines; 2 Division of Basic Science and Translational Research, Department of Obstetrics and Gynecology, The University of Texas Medical Branch at Galveston, Galveston, TX 77555, USA; ra2menon@utmb.edu; 3 Department of Biochemistry and Molecular Biology, University of the Philippines Manila, Manila 1000, Philippines; grafebyu@gmail.com; 4 Department of Obstetrics and Gynecology, Philippine General Hospital, University of the Philippines Manila, Manila 1000, Philippines; mdamosco@up.edu.ph

**Keywords:** bibliometrics, environmental toxicants, pollution, preterm birth

## Abstract

Preterm birth remains a problem globally, as multiple factors contribute to its etiology and pathogenesis. One such factor is the exposure to environmental toxicants, in which recent literature has described contributory roles in disease progression. This study aims to show research trends and collaborations in papers related to environmental toxicants and preterm birth through a bibliometric analysis to determine hot spots for research as well as to identify already established themes that can point to policy making and development. Using the Scopus database, we were able to identify 956 original research articles from 72 countries between 1955 and 2021; bibliographic information was exported, analyzed, and visualized using Bibliometrix and VOSviewer. There was an annual growth of research and reporting in this area, which significantly increased within the last two decades. The top countries that have published on this topic include the USA (*n* = 343), China (*n* = 103), and Australia (*n* = 43), with strong international collaboration in reports from China. Top journals for publication include *Environmental Research* (*n* = 53), *Environmental Health Perspectives* (*n* = 47), and *Environment International* (*n* = 46). Previous literature focused on establishing toxicants that are significantly associated with preterm birth, with current research focusing on molecular mechanisms of environmental toxicants. Overall, our bibliometric analysis gives a scoping view of the existing research landscape in terms of environmental health and preterm birth.

## 1. Introduction

Preterm birth remains a persistent problem in the world, with annual rates ranging from 5–18% of all infants born globally [1]. Although multiple etiologies have already been proposed that contribute to preterm delivery, only a few of these factors have been well studied and reported due to greater research into those aspects. For instance, infection is well established to lead to inflammation that may lead to early spontaneous preterm birth [2]. Intrauterine bleeding activates thrombin and leads to cytokine and chemokine influx that induces myometrial contractions, resulting in preterm birth [3]. Uterine distension, either via polyhydramnios, multiple gestations, or congenital abnormalities, induces stretch receptor-mediated inflammatory responses that may also lead to preterm birth [4].

One of the emerging risk factors for preterm birth is environmental toxicants. The catalog comprises a vast array of chemicals, ranging from organic pollutants (persistent, non-persistent, and water disinfectants), metals (lead, arsenic, mercury, cadmium), and air pollutants (gases, particulate matter, polycyclic aromatic hydrocarbons, volatile organic compounds, and tobacco smoke) [5]. In varying degrees, these compounds have been shown to lead to increased rates of premature delivery, from small to large positive associations. As examples, exposure to high levels of organochlorine pesticides such as DDT were linked to higher risks of preterm delivery [6,7]. Multiple reports on air pollution and particulate matter showed strong associations with preterm birth, especially for women with prior preterm birth [8,9,10]. Metal exposure, especially lead and cadmium, appear to increase risks of preterm birth as well [11].

More studies are still needed to understand the mechanisms of the environment and toxicant exposure on pregnancy outcomes. Analysis of a two-decade research output on preterm birth demonstrated that research regarding “Public, Environment, and Occupational Health” and preterm birth only had a marginal increase in the share of preterm birth research, from 0.6% in 1997 to 1.0% in 2016 [12]. A combination of industrialization and gaps in local policies on pollution would naturally lead to increases in exposure of vulnerable populations, specifically pregnant women, to environmental toxicants [13]. Therefore, scoping the literature regarding the progress in research in this field can potentially determine knowledge gaps to address through future research as well as identify already established themes that can guide policy making and development.

Bibliometric analysis provides a powerful avenue to view how overall research in a field progresses over time. Tracking multiple quantitative parameters in terms of journal, author, country, keywords, and article citations through bibliometrics enables researchers to develop conceptual, intellectual, and social structures that can be analyzed [14]. Multiple softwares are available as open sources for bibliometric analysis. One tool that can be used is Bibliometrix, a science mapping software that runs as an R package. It runs a partner web interface app, Biblioshiny, that allows running of command line functions in a more convenient, user-friendly manner [15]. Visualization tools for keyword co-occurrence and author co-authorship networks can be done natively, but one application commonly used is the Java-run VOSviewer [16]. Using these two tools, we set out to perform bibliometric analysis regarding research on environmental toxicants and preterm birth.

## 2. Materials and Methods

### 2.1. Study Selection

An extensive systematic search of literature was performed using the Scopus database [17]. We utilized the search argument [(“environmental toxi*” OR “toxicants” OR “pollut*” OR metal* OR arsenic OR cadmium OR mercury OR smoke OR particulate OR dde OR pcb OR hch OR hcb OR bisphenol OR phthalate OR phenyl OR pesticide OR dioxin OR pfc OR pfoa OR pfos OR ddt OR pah ) AND (“preterm birth” OR “premature birth” OR “premature delivery” OR “decreased gestational age”)]. We limited our search to full-text original articles as well as open access articles published from 1955–2021. We excluded review articles and letters/notes. An electronic search was performed on 29 August 2021.

### 2.2. Data Collection

A total of 956 bibliographic records were downloaded from the Scopus database. The following metadata were obtained: authors, title, year of publication, source title, citation count, source type, DOI, affiliations, publisher, editors, language, correspondence address, abstract, author keywords, index keywords, and references. The 2021 impact factor was obtained from the master journal list of Clarivate [18].

### 2.3. Statistical Analysis

Statistical analysis and graph generation were undertaken using the Biblioshiny package of the Bibliometrix package version 3.1 of R version 4.1 (R Studio version 1.4.1717) [15]. The visualization of co-occurrences of keywords related to environmental toxicants and preterm birth as well as co-authorship networks were conducted using VOSviewer version 1.6.16 [16].

## 3. Results

**Figure 1:**
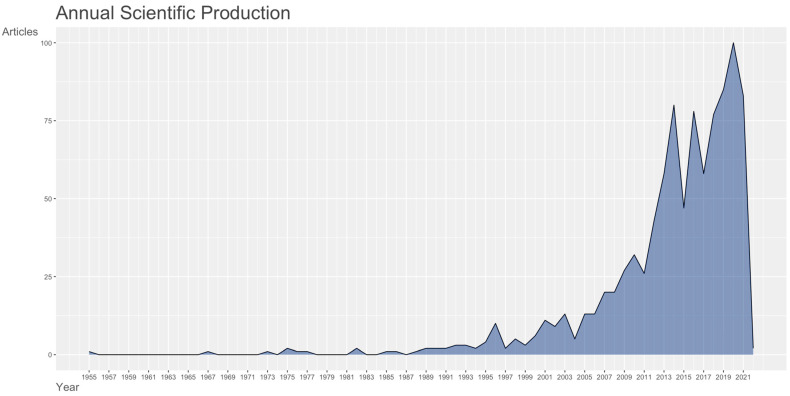
Annual scientific production for articles on environmental toxicants and pollutants and preterm birth.

We included a total of 908 journal articles spanning from 1955 to 2021, obtained from 385 source documents. The average annual growth rate for publications regarding preterm birth and environmental toxicants is estimated at 1.63%, and the trend is shown in Figure 1.

The most impactful journal article was by Michael Brauer, entitled “A Cohort Study of Traffic-Related Air Pollution Impacts on Birth Outcomes “ (local citation (LC) = 71, global citation (GC) = 419, LC/GC ratio = 16.95). Two of the three most impactful journal articles belonged to Beate Ritz, entitled “Effect of air pollution on preterm birth among children born in Southern California between 1989 and 1993” (LC = 67, GC = 263 LC/GC ratio = 25.48) and another study entitled “Ambient air pollution and preterm birth in the environment and pregnancy outcomes study at the University of California, Los Angeles” (LC = 60, GC = 263, LC/GC ratio = 22.81). Data are summarized in Table 1.

### 3.1. Publications by Journals

**Figure 2:**
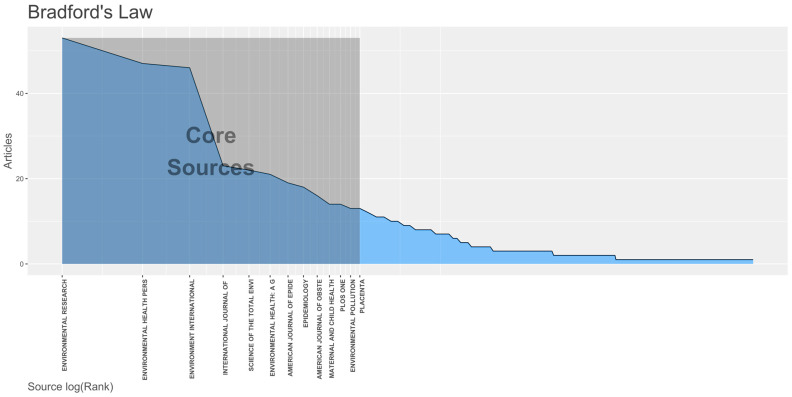
Core sources for publications on environmental toxicants and pollutants and preterm birth.

The top journals with the highest number of published research were *Environmental Research* (*n* = 53 articles, total citations (TC) = 1289 h-index = 21, impact factor (IF) = 6.498), followed by *Environmental Health Perspectives* (*n* = 47, TC = 4662, h-index = 35, IF = 9.031), and then *Environment International* (*n* = 46, TC = 1215, h-index = 20, IF = 9.621). The top 10 journals also comprise core sources among 369 journals publishing about the topic as shown in Figure 2, along with *PLoS ONE, Environmental Pollution,* and *Placenta*. Among the top 10 journals, six of these are relevant to environmental themes, two emphasize epidemiological relevance (*American Journal of Epidemiology, Epidemiology*), while two cater to obstetrics, gynecology, and child health (*American Journal of Obstetrics* and *Gynecology, Maternal and Child Health*). The results are summarized in Table 2.

### 3.2. Publication by Authors

**Figure 3:**
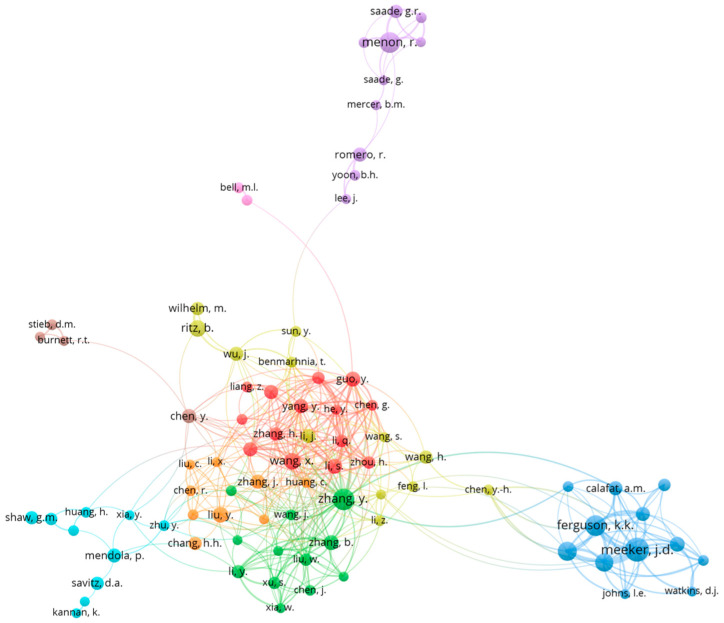
Co-authorship network showing multiple clusters of co-authors, with centralization among a majority of Chinese authors.

The top authors who have published the highest number of papers were John Meeker of the School of Public Health, University of Michigan (*n* = 27, fractional authorship (FA) = 2.328, h-index = 18, TC = 321), Ramkumar Menon of the University of Texas Medical Branch (*n* = 21, FA = 3.94, h-index = 12, TC = 560 ), and Kelly Ferguson of the US National Institute of Environmental Health Sciences (NIEHS) (*n* = 20, FA = 3.26, h-index = 16, TC = 1097). Data are shown in Table 3. Only around 1.98% of all authors have published at least four papers, with 81.4% of all authors only publishing a single paper. Co-authorship network visualization reveals multiple clusters of co-authorship with major clustering around authors from China, as seen in Figure 3.

### 3.3. Publications by Institutions

The top institutions publishing regarding the topic of interest include the University of California, California, USA (*n* = 97), followed by the Harvard University (Harvard Medical School and Harvard TH Chan School of Public Health), Massachusetts, USA (*n* = 91), and the University of Michigan (University of Michigan and University of Michigan School of Public Health), Michigan, USA (*n* = 67). Two of the 10 institutions are outside the United States (Peking University, Beijing, China and Sun-Yat Sen University, Guangzhou, China). Results are summarized in Table 4.

### 3.4. Publications by Countries

**Figure 4:**
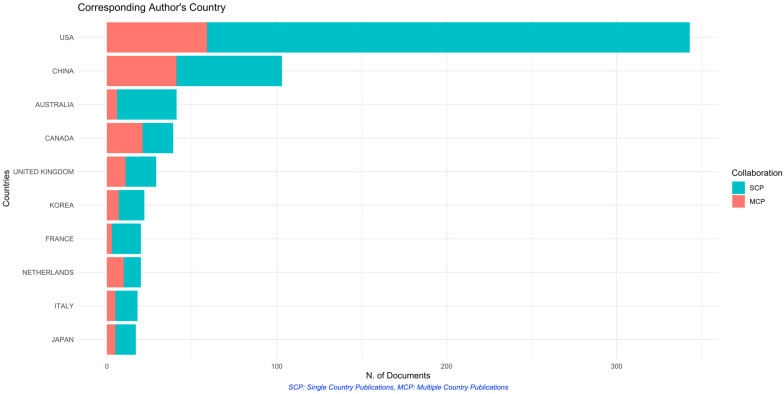
Top countries publishing on environmental toxicants and pollutants and preterm birth.

The top countries that have published the highest number of papers include the USA (*n* = 343, single country publications (SCP) = 284, multiple countries publication (MCP) = 59, MCP ratio = 0.1720, TC = 13798), China (*n* = 103, SCP = 62, MCP = 42, MCP ratio = 0.398, TC = 1848), and Australia (*n* = 41, SCP = 35, MCP = 216 MCP ratio = 0.0.1463, TC = 923). Results are shown in Table 5 and Figure 4.

### 3.5. Keyword Visualization

**Figure 5:**
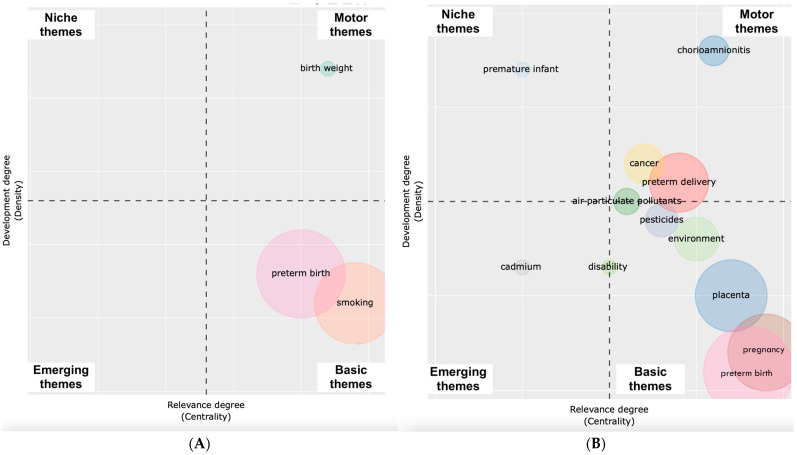

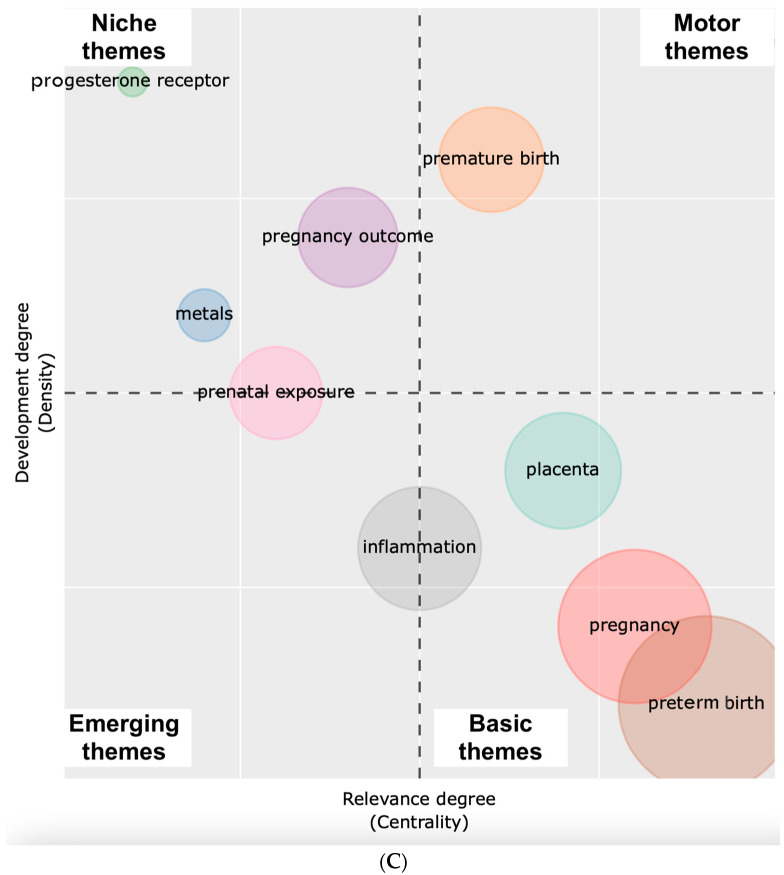
Thematic evolution of author keywords from (**A**) 1973 to 2000, (**B**) 2000–2012, and (**C**) 2013–present.

We identified three time periods, from 1955–2000, 2001–2012, and 2013–present, as we observed that there was a spike in citations for 2000 (number of references = 453) due to the publication of Ritz et al. (“Effect of air pollution on preterm birth among children born in Southern California between 1989 and 1993”) and the peak of publication references was reached at 2012 (number of references = 2304). We analyzed thematic evolution of author keywords over these three time periods through thematic maps shown in Figure 5. From 1955–2000, there were two transversal themes (“preterm birth” and “smoking”) with a single well-developed theme (“birth weight”). From 2001–2012, we saw a burst of motor and transversal themes. Highly developed themes were research involving “development”, “chorioamnionitis”, “air pollutants” and “cancer”. Basic themes involved research on “pesticides”, “environment”, “placenta”. “Birth weight” and “preterm birth” converged into a single basic theme of “preterm birth”. From 2013–present, “pesticides” diverged into research on “prenatal exposure” and “premature birth”, “placenta” diverged into two additional themes on “progesterone receptor” and “inflammation”. Research on “cadmium” converged with “placenta”. “Preterm birth” and “preterm delivery” diverged into “preterm birth”/”premature birth” and “pregnancy outcomes”. Studies on “metals” and “progesterone receptor” appeared and were relegated as niche themes.

**Figure 6:**
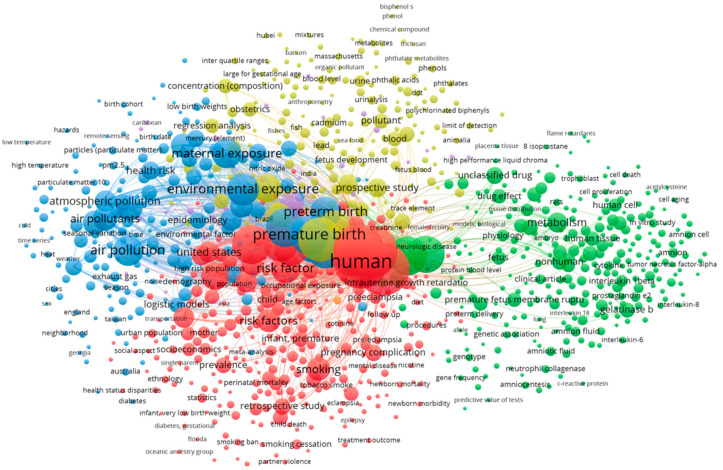
Co-occurrence network showing four clusters of keywords relating to human studies (red), air and particulate matter pollution (blue), environmental pollutants (yellow), and molecular studies on non-human and tissue/cellular samples (green).

Co-occurrence analysis was also undertaken, as shown in Figure 6. Overall, four clusters of keywords were observed. These were designated as relating to human studies (red), air and particulate matter pollution (blue), environmental pollutants (yellow), and molecular studies on non-human and tissue/cellular samples (green).

## 4. Discussion

Although there is a multitude of studies that explore the association between environmental toxicants and preterm birth, no analysis has been undertaken yet that identifies global research output and trends in this field. This paper shows also the main contributors to the field of study, as well as publication preferences and regional output.

Early research has focused on effects of smoking on preterm birth and birth weight. A meta-analysis published in 2000 demonstrated a 1.27 pooled estimate risk for preterm delivery among maternal smokers when compared to non-smokers [19]. Although multiple papers have been published regarding preterm birth and smoking, only a few papers have delved into the component-specific mechanisms responsible for adverse outcomes [20]. In our thematic analysis, preterm birth slowly merged with smoking over the time slices, fitting so since it has already been established as a risk factor for preterm birth. Meanwhile, research on environmental pollutants, such as persistent organic pollutants and metals contaminants, occurred near the year 2000, and the following decade saw an increase in papers publishing about these pollutants. 

Particularly interesting is air pollution, as papers about its association with preterm birth along with particulate matter were published in the 2000s. The following decade saw cohort studies by Ritz and Brauer on the impact of air pollution, and our analysis showed that these articles have been the most impactful in terms of local as well as global citations [8,9,10]. Ritz et al. observed a preterm birth risk ratio of 1.20 for those exposed to ambient levels of particulate matter (PM) <10 μm in aerodynamic diameter (PM10), and elevated risks as well for those exposed to carbon monoxide and PM2.5 [8,9]. Brauer observed associations with PM2.5 and preterm births <37 weeks, with other air pollutants associated with preterm birth at <30 weeks [10]. These papers essentially established air pollution as a risk factor, and over the years it converged as well with preterm birth in our thematic analysis.

We observed a peak in publication in this area within the last 10 years, especially in 2014, 2016, and 2020. During the last decade, the bigger themes included “placenta” and “inflammation” as well as the niche theme “progesterone receptor”, indicating that research now tend to delve into the mechanisms of preterm birth with exposure to environmental toxicants. Time sensitivity has also been explored in this period, since “prenatal exposure” also appeared. Most of the research also involve “pregnancy outcomes”, perhaps indicating that the latest publications also examine postpartum impacts such as birth weight and infant disabilities. From research on cadmium in the second time period, now the latest research includes other “metals” as a niche theme and may relate to molecular mechanisms as well.

There is also a regional imbalance in terms of global research output. The top countries in which most of the research has been done are highly industrialized countries such as the United States and China. Top authors in the field also are stationed in the United States, with Drs Meeker and Ferguson publishing mostly on epidemiological evidence of preterm birth and adverse pregnancy outcome association with multiple environmental pollutants. Dr Menon’s work is primarily focusing on mechanistic studies on how pollutants such as cigarette smoke, PBDE, and BPA/BPS cause preterm birth [21,22,23]. Interestingly, articles published in China had a higher intensity of international collaboration compared to their American counterparts. Also, most researchers as observed in the co-authorship network plot tend to concentrate on Chinese authors, with outside clusters of American or other European/North American origin. In particular, air pollution is widely explored in China as there are higher pollution levels along with genetic differences in comparison to other populations [24,25]. In the United States, air pollution has also been widely explored along with other environmental pollutants such as pesticides and metals/metalloids, among others [5]. Indeed, economic development and environmental pollution are two inseparable themes, as the increase in demand for infrastructure resources along with increased consumption of pollution-intensive resources may lead to pollution especially in developing countries [26]. Although considerable evidence can be obtained from developed countries, there must also be representative research in developing countries in order to fully estimate the impact of these environmental pollutants in terms of adverse pregnancy outcomes. However, research endeavors on this topic may be limited in these countries depending on the country’s health research agenda, local institutions that cater to the research topic, and also by the individual country’s research spending [27]. Developing countries such as India, where pollution-related health issues are rampant, are yet to report on environmental impacts on preterm birth. 

We also observed that most authors tend to publish in high-impact journals on environmental health. Although *Environmental Research* has the highest number of papers, *Environmental Health Perspectives* has a much higher h-index and total citations thus making it the most impactful journal. The latter is also published by the National Institutes of Health (NIH)/NIEHS, and may play a role in its impact within the local community [28]. Although the inclusion of two obstetrics and child health journals within the top 10 is not surprising, more interesting would be the inclusion of two epidemiological research journals. Thus, it can be inferred that this research area is not only relegated to purely clinical aspects, but is also a pressing public health concern that needs an epidemiological assessment and action [29].

The strength of this study involves the use of two different software for bibliometric analysis and network visualization. Bibliometrix, among other bibliometric tools for science mapping analysis, stands out with its intuitive interface as well as the incorporation of powerful features, such as evolution analysis, performance analysis, and reference spectroscopy; VOSviewer on the other hand allows for a more intensive network visualization through three-step co-occurrence matrix creation, zoom and scroll option, and smart labeling to avoid overlaps [30].

One limitation of this study is that only a single database was used for the literature search. However, by using the Scopus database, we are confident that the most relevant literature in the field has been captured since Scopus allows for greater coverage for citation analysis and journal range compared to other big databases such as Web of Science, Pubmed, or Google Scholar [31]. Future research may combine our results with literature retrieved from other databases in order to have a comprehensive look at research trends in environmental pollution and preterm birth. Another limitation of this study is that only full-text research articles were included, and review articles, editorials, and position/hypothesis papers were excluded. Nonetheless, this paper provides a robust overview of the research progress in terms of environmental toxicants and preterm birth.

## 5. Conclusions

Our bibliometric analysis demonstrates the research trends and research collaborations among researchers in the field of environmental toxicants and preterm birth since 1955. Currently, research in this subject matter seems to expand beyond epidemiological association studies and gradually delve into mechanistic studies. Although annual production in this field has grown substantially within the last two decades, overall growth is still small despite massive industrialization and increased exposure to environmental pollution. The research has evolved from establishing significant environmental toxicants to exploring the molecular mechanisms of action of these pollutants. Most studies were from developed countries and further representation of developing countries is needed for researchers to gain a better picture of the current state of science and evidence related to preterm birth. Increasing research involvement in such countries may help form local and global policies on pollution control in order to reduce the incidence of preterm birth.

## Figures and Tables

**Table 1:** Most cited documents on environmental toxicants and pollutants and preterm birth.

Document	Title and DOI	Year	Local Citations	Global Citations	LC/GC Ratio (%)	Normalized Local Citations	Normalized Global Citations
Brauer M, 2008, *Environ Health Perspect*	“A Cohort Study of Traffic-Related Air Pollution Impacts on Birth Outcomes”10.1289/ehp.10952	2008	71	419	16.95	8.40	5.74
Ritz B, 2000, *Epidemiology*	“Effect of air pollution on preterm birth among children born in Southern California between 1989 and 1993”10.1097/00001648-200009000-00004	2000	67	263	25.48	3.72	1.52
Ritz B, 2007, *Am J Epidemiol*	“Ambient air pollution and preterm birth in the environment and pregnancy outcomes study at the University of California, Los Angeles”10.1093/aje/kwm181	2007	60	263	22.81	7.14	3.34

DOI: Digital object identifier, LC/GC Ratio: Local citations/Global citations ratio.

**Table 2:** Top journals that published regarding environmental toxicants and pollutants and preterm birth.

Sources	Articles	H-Index	TC
*Environmental Research*	53	21	1289
*Environmental Health Perspectives*	47	35	4662
*Environment International*	46	20	1215
*International Journal of Environmental Research and Public Health*	23	8	186
*Science of the Total Environment*	22	12	413
*Environmental Health: A Global Access Science Source*	21	14	949
*American Journal of Epidemiology*	19	17	1274
*Epidemiology*	18	16	1366
*American Journal of Obstetrics And Gynecology*	16	13	768
*Maternal and Child Health Journal*	14	8	300

TC: Total citations.

**Table 3:** Top authors that published regarding environmental toxicants and pollutants and preterm birth.

Authors	Articles	Articles Fractionalized	H-Index	TC
Meeker JD	27	4.07	18	522
Menon R	21	3.94	12	560
Ferguson KK	20	3.26	16	1097
McElrath TF	17	2.94	14	890
Mukherjee B	16	2.52	13	733
Ritz B	13	3.39	12	1664
Cordero JF	11	1.24	6	272
Calafat AM	11	1.02	8	502
Mendola P	10	2.49	6	12
Cantonwine DE	9	1.24	9	521

TC: Total citations.

**Table 4:** Top institutions that published regarding environmental toxicants and pollutants and preterm birth.

Affiliations	Articles
University of California	97
Harvard University	91
University of Michigan	67
Emory University	30
Wayne State University	25
Yale University	25
Peking University	20
National Institute of Environmental Health Sciences	19
University of South Florida	19
Sun Yat-Sen University	18

**Table 5:** Top countries that published regarding environmental toxicants and pollutants and preterm birth.

Country	Articles	TC	SCP	MCP	MCP Ratio
USA	343	13,798	284	59	0.1720
China	103	1848	62	41	0.3981
Australia	41	923	35	6	0.1463
Canada	39	2497	18	21	0.5385
United Kingdom	29	1181	18	11	0.3793
Korea	22	479	15	7	0.3182
France	20	263	17	3	0.1500
Netherlands	20	1366	10	10	0.5000
Italy	18	445	13	5	0.2778
Japan	17	555	12	5	0.2941

TC: Total citations, SCP: Single country publications, MCP: Multiple countries publications.

## Data Availability

Not applicable.
